# Supplementary material for: Multi‐dataset identification of innovative feature genes and molecular mechanisms in keratoconus
Source: J Cell Mol Med. 2024 Sep 19;28(18):e70079. doi: 10.1111/jcmm.70079 (PMC11412914; doi:10.1111/jcmm.70079)
Supplement: Supplementary file 2 — Material S2. [file JCMM-28-e70079-s002.zip › 7.GSEA analysis/3.DBNDD1/KEGG_PROXIMAL_TUBULE_BICARBONATE_RECLAMATION.html]

Details for gene set KEGG\_PROXIMAL\_TUBULE\_BICARBONATE\_RECLAMATION[GSEA]

|  || Dataset | DBNDD1.group.cls#h\_versus\_l |
| Phenotype | group.cls#h\_versus\_l |
| Upregulated in class | l |
| GeneSet | KEGG\_PROXIMAL\_TUBULE\_BICARBONATE\_RECLAMATION |
| Enrichment Score (ES) | -0.4013825 |
| Normalized Enrichment Score (NES) | -1.2300249 |
| Nominal p-value | 0.16536964 |
| FDR q-value | 0.7073003 |
| FWER p-Value | 0.985 |
Table: GSEA Results Summary

  

Fig 1: Enrichment plot: KEGG\_PROXIMAL\_TUBULE\_BICARBONATE\_RECLAMATION      
 Profile of the Running ES Score & Positions of GeneSet Members on the Rank Ordered List

  

| SYMBOL | TITLE | RANK IN GENE LIST | RANK METRIC SCORE | RUNNING ES | CORE ENRICHMENT || 1 | ATP1B4 | na | 2619 | 0.267 | 0.0266 | No |
| 2 | SLC38A3 | na | 6472 | 0.137 | -0.0319 | No |
| 3 | ATP1B1 | na | 9417 | 0.080 | -0.0863 | No |
| 4 | SLC25A10 | na | 9550 | 0.077 | -0.0605 | No |
| 5 | PCK1 | na | 9869 | 0.073 | -0.0417 | No |
| 6 | ATP1A3 | na | 11874 | 0.048 | -0.0810 | No |
| 7 | GLS2 | na | 12449 | 0.043 | -0.0811 | No |
| 8 | GLUD1 | na | 15404 | 0.020 | -0.1586 | No |
| 9 | SLC9A3 | na | 15933 | 0.016 | -0.1676 | No |
| 10 | AQP1 | na | 18523 | -0.001 | -0.2418 | No |
| 11 | MDH1 | na | 19861 | -0.012 | -0.2758 | No |
| 12 | GLS | na | 21167 | -0.023 | -0.3045 | No |
| 13 | ATP1A4 | na | 21341 | -0.025 | -0.2998 | No |
| 14 | GLUD2 | na | 23554 | -0.050 | -0.3443 | No |
| 15 | ATP1B3 | na | 25535 | -0.079 | -0.3711 | Yes |
| 16 | ATP1A1 | na | 25979 | -0.087 | -0.3507 | Yes |
| 17 | ATP1B2 | na | 26095 | -0.089 | -0.3199 | Yes |
| 18 | CA4 | na | 28858 | -0.153 | -0.3410 | Yes |
| 19 | FXYD2 | na | 29524 | -0.173 | -0.2940 | Yes |
| 20 | ATP1A2 | na | 30814 | -0.214 | -0.2492 | Yes |
| 21 | SLC4A4 | na | 30923 | -0.218 | -0.1689 | Yes |
| 22 | PCK2 | na | 32336 | -0.284 | -0.1011 | Yes |
| 23 | CA2 | na | 34111 | -0.445 | 0.0178 | Yes |
Table: GSEA details [plain text format]

  

Fig 2: KEGG\_PROXIMAL\_TUBULE\_BICARBONATE\_RECLAMATION      
 Blue-Pink O' Gram in the Space of the Analyzed GeneSet

  

Fig 3: KEGG\_PROXIMAL\_TUBULE\_BICARBONATE\_RECLAMATION: Random ES distribution      
 Gene set null distribution of ES for **KEGG\_PROXIMAL\_TUBULE\_BICARBONATE\_RECLAMATION**
